# Rapid prototyping of a polymer MEMS droplet dispenser by laser-assisted 3D printing

**DOI:** 10.1038/s41378-023-00559-3

**Published:** 2023-07-04

**Authors:** Rémi Courson, Oleksii Bratash, Ali Maziz, Cloé Desmet, Ricardo Alvarado Meza, Loïc Leroy, Elodie Engel, Arnaud Buhot, Laurent Malaquin, Thierry Leïchlé

**Affiliations:** 1 LAAS-CNRS, Université de Toulouse, CNRS, 31400 Toulouse, France; 2 Université Grenoble Alpes, CNRS, CEA, IRIG, SyMMES, 38000 Grenoble, France; 3 Georgia Tech−CNRS International Research Laboratory, Atlanta, GA 30332 USA

**Keywords:** Electrical and electronic engineering, Biosensors, Nanofabrication and nanopatterning

## Abstract

In this work, we introduce a polymer version of a previously developed silicon MEMS drop deposition tool for surface functionalization that consists of a microcantilever integrating an open fluidic channel and a reservoir. The device is fabricated by laser stereolithography, which offers the advantages of low-cost and fast prototyping. Additionally, thanks to the ability to process multiple materials, a magnetic base is incorporated into the cantilever for convenient handling and attachment to the holder of a robotized stage used for spotting. Droplets with diameters ranging from ∼50 µm to ∼300 µm are printed upon direct contact of the cantilever tip with the surface to pattern. Liquid loading is achieved by fully immersing the cantilever into a reservoir drop, where a single load results in the deposition of more than 200 droplets. The influences of the size and shape of the cantilever tip and the reservoir on the printing outcome are studied. As a proof-of-concept of the biofunctionalization capability of this 3D printed droplet dispenser, microarrays of oligonucleotides and antibodies displaying high specificity and no cross-contamination are fabricated, and droplets are deposited at the tip of an optical fiber bundle.

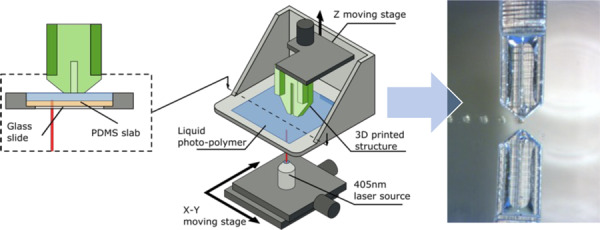

## Introduction

Surface functionalization is an important step for the realization of biosensors and microarrays. To address these applications, functionalization tools should enable the deposition of biological entities at specific locations with micrometric feature sizes. Among the eligible patterning techniques, numerous direct writing methods enable the patterning of surfaces from scratch with different materials^1^. Direct patterning tools that are based on the delivery of liquids can be classified into contact and noncontact methods^2^. Noncontact techniques, such as ink-jet printing^3^ and acoustic droplet ejection^4^, reduce the hazards of substrate/material damage and contamination; however, they are prone to clogging issues and satellite droplet production. In addition, these techniques are usually characterized by large droplet flow rates, which is advantageous for large-scale printing but consumes a high volume of reagents, posing an issue for some applications. Conversely, contact methods rely on drop delivery upon direct contact of the tool with the surface to pattern. Metal pins and styluses, designed for the delivery of nL volumes, are common tools used to fabricate microarrays^5^. Microarray density and resolution can be increased by dip-pen technology, which creates nanoscale biological patterns by transferring molecules absorbed on an atomic force microscope (AFM) tip via the liquid meniscus that forms between the tip and the surface in a high-humidity environment^6^. Nanofountain probes use modified AFM tips with micromachined enclosed reservoirs^7^ or focused-ion-beam milled open reservoirs^8^ to increase the deposited volume to the aL scale; nevertheless, similar to all AFM-based techniques, these are rather high cost and low throughput.

To fill the gap size existing between the aforementioned direct contact technologies (metal pins and nanofountain probes that create patterns typically larger than 100 µm and smaller than 1 µm, respectively), we have previously presented a droplet deposition tool called Bioplume based on the local delivery of liquid that creates controlled micrometer size patterns^9^. This dispensing tool consists of silicon cantilevers bearing open microfluidic channels. Drop deposition occurs by liquid transfer from the cantilever to the surface to pattern upon direct contact between the cantilever tip and the surface. The patterned features typically range from 7 μm to 250 μm in diameter, depending on the spotting time and surface wettability^10^, and droplets with diameters of 2 µm are achieved using a cantilever version that integrates a nanotip with a curvature radius of 100 nm^11^. Since the cantilever microchannel is partially open and the liquid–air surface-area-to-volume ratio is large, the liquid loaded on the cantilever is prone to quick evaporation. The same trend is true for the spotted droplets. Hence, glycerol or dimethyl sulfoxide (DMSO) is added to the spotting solution, which is either water or biological buffers, to reduce the evaporation rate; this strategy is commonly used with microarray spotters^12^. This tool is designed to functionalize MEMS biosensors^13^ and to fabricate DNA, protein, and cell microarrays^14,15^, where multiplexing is achieved using off chip cartridges; multiplexing can eventually be performed with on-chip ink reservoirs^16^. This tool has been used to create patterns of self-assembled nanoparticles^17^ and to deposit polymer droplets used as optical lenses^18,19^ or as synthetic bioreceptors^20^. Bioplume cantilevers are fabricated using microfabrication methods in a clean room environment. The use of microelectronic-derived technologies allows the realization of high-resolution micrometric feature sizes. Moreover, piezoresistors can easily be integrated onto cantilevers by silicon doping and then be used as force sensors for closed-loop controlled spotting^9^. Finally, this technology offers the additional advantage of inducing electrochemical reactions within the droplets during deposition by means of a metal electrode integrated to the fluidic channel^21^. This feature is used for the fabrication of microarrays using pyrrole electrochemistry^22^; additionally, this electrode is used to induce electrowetting-assisted loading^23^ and to control the spot size during drop deposition^24^. However, the use of microfabrication techniques in a clean room environment has several disadvantages. In addition to being very expensive and time-consuming, another drawback is the lack of flexibility in design adjustment: changing the geometry and size of the cantilevers implies the modification of many photolithography masks and the execution of a lengthy fabrication process.

The field of additive manufacturing (3D printing) has experienced considerable growth in recent years, and we can now print 3D objects with complex shapes from various materials (metals, ceramics, plastics, hydrogels, etc.) for innovative applications ranging from biomedicine to aeronautics^25^. The recent improvements in 3D stereolithography resolution^26–28^ have permitted the extension of additive manufacturing technologies to the fabrication of microfluidic devices with complex geometries^29^ and microsystems with resolutions down to the microscale^30–32^. These developments have led to the emergence of a 3D printed version of a microfluidic probe that enables local chemical reactions in a solution by injecting and aspirating fluids with two microapertures^33^. The combination of different 3D additive manufacturing techniques, stereolithography and two-photon polymerization, is also used to directly print ready-to-use microfluidic AFM cantilever probes for cell manipulation^34^.

In this work, we propose a polymer version of the droplet dispensing tool Bioplume realized by 3D stereolithography that differs from other spotter approaches using an open microfluidic channel and reservoir for efficient device washing. Just as for 3D printed MEMS and microfluidic devices, the advantages of using 3D printing for this specific application are numerous. 3D printing allows for facile, affordable and fast (<1 h) manufacturing processes, thus enabling rapid prototyping with design flexibility, where various shapes and dimensions are easily accessible. Hence, we explore various cantilever designs derived from the original Bioplume device, and we study the influences of the sizes and shapes of the cantilever tip and the reservoir on the printing outcome. We have taken advantage of the ability to process multiple materials to incorporate a magnetic base into the cantilever for convenient handling and attachment to the holder of a robotized stage used for spotting. Finally, since the cantilever is made from a biocompatible polymer with adequate wettability that exhibits a relatively low Young’s modulus vs. silicon, biomolecules are printed via direct contact without damaging the patterned surface. As a result, we present a spotting device that can print up to 200 drops in a single load with a maximum speed of 2 droplets/cantilever/second. The drop diameter ranges from ∼50 µm to >300 µm, according to the contact time and surface wettability. As a proof-of-concept of the biofunctionalization capability of this 3D printed droplet dispenser, microarrays of two different oligonucleotides with preserved biological functionality and high specificity are fabricated. Two fluorescent antibodies are sequentially spotted without observed cross-contamination to highlight the simplicity and efficiency of the washing protocol used between the loading steps.

## Experimental method

### Design and fabrication of the polymer cantilevers by 3D printing

**Fig. 1:**
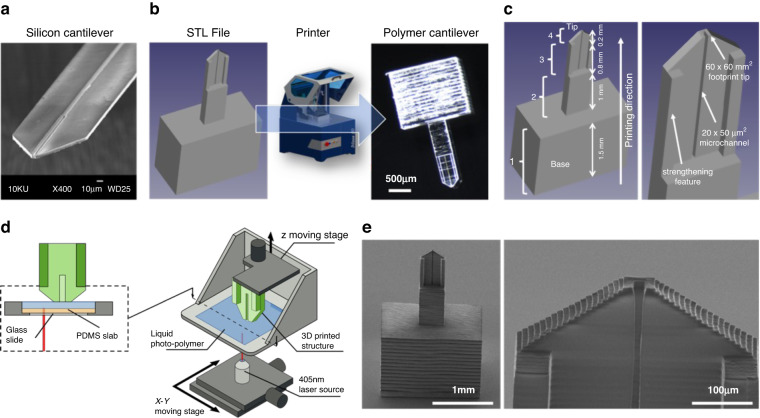
Fabrication of the polymer droplet dispenser. **a** SEM image of a previously fabricated Bioplume silicon cantilever that inspired the design of the polymer cantilever. **b** Illustration of the fabrication process of a polymer cantilever by stereolithography from an STL file design. **c** Schematics of a V1 cantilever indicating the various parts of the device along with their dimensions and the 4 regions fabricated using different printing parameters. **d** Schematics of the stereolithography apparatus and process where the cantilever is fabricated via layer-by-layer polymerization of a liquid photopolymer using a laser beam. **e** SEM images of a 3D-printed V1 cantilever showing the cuboid base, the cantilever, the cantilever tip and the microfluidic channel

The design of the polymer cantilevers was directly inspired by the silicon-based Bioplume device^9^. The original device consisted of multiple 5-µm-thick, 120-µm-wide and 1.5-mm-long microcantilevers fabricated onto a silicon chip, each including a 4-µm-wide microfluidic channel opening onto the tip (Fig. 1a). The present design was carried out using 3D CAD software, resulting in a standard tessellation language (STL) file, and cantilevers were subsequently fabricated by stereolithography (Fig. 1b) from the biocompatible DS-3000 photopolymer (DWS, Italy)^35^ using a Dilase 3D printer (Kloé SA, France)^27^. For convenient manipulation with tweezers, the cantilever devices included a cuboid polymer base and were directly fabricated onto a glass or silicon substrate, from which they could be easily removed after fabrication.

We designed and fabricated cantilevers of various shapes, features and dimensions. The reference sample (V1) consisted of a 2-mm-long and 500-µm-wide cantilever (Fig. 1c). The cantilever was 200-µm-thick and 60-µm-thick at its base and at its extremity, respectively. The cantilever exhibited a 45° angle sharpened extremity leading to a 60 × 60 µm cross-sectional tip. A one-sided open, 1-mm-long, 20-µm-wide microfluidic channel was integrated onto the cantilever and opened at its tip with a cross section of 50 × 20 µm. The total sample volume (including the cuboid base) was 2 × 1 × 3.5 mm^3^. Due to the polymer having a lower Young’s modulus than silicon (1–3 GPa^35^ vs. 165 GPa), strengthening features consisting of two 60-µm-thick, 80-µm-wide and 800-µm-long beams were added to the surface of the cantilever to avoid deformations induced by the internal stress from layer-to-layer polymerization.

The cantilevers were fabricated by stereolithography, which is a layer-by-layer polymerization process induced by a laser beam (Fig. 1d), using a Dilase 3D HR equipped with a 405-nm laser source working at 50 mW that exhibited an x/y resolution of 5 µm, a z resolution ranging from 5 to 100 µm, a polymer roughness of 2 µm, and a maximum writing speed of 50 mm/s for a maximum sample size of x,y,z = 10 × 10 × 5 cm^3^. The 3D printing process was conducted in several steps. The first layer (100 µm between the build table and the bottom of the tank containing the photoresist) was fabricated at 100% laser power and a 20 mm/s writing speed to ensure good adhesion of the sample onto the supporting silicon or glass substrate and to guarantee parallelism between the first constructed layer and the bottom of the tank. Then, four regions were defined according to the design of the cantilever, as shown in Fig. 1c (increasing z), each associated with a different set of parameters (Table 1). For each region, the slice thickness was selected to accurately reproduce the cantilever design, and the printing speed and power were adjusted to provide the maximum printing speed while avoiding overexposure effects. The base of the cantilever (region 1; from 0 to 1.5 mm) was printed with the maximum slice thickness allowed by the printer (100 µm). For region 2 (from 1.5 to 2.5 mm), the slice value was decreased to 50 µm to maintain a high printing velocity while limiting the edge roughness. Parameters were further modified to print the end of the cantilever in regions 3 and 4 that included the 20 µm wide microchannel. For region 3, the slice thickness was adjusted to 20 µm to avoid clogging the microchannel. The laser power and writing speed were reduced to 35% and 6 mm/s, respectively, to compensate for the resulting dose increase. Finally, the slice thickness in region 4 was further reduced to 10 µm to accurately reproduce the tapered shape of the cantilever. A laser power of 35% and a speed of 5 mm/s were the optimum parameters to reach the expected dimensions of the pointed cantilever shape while preserving the maximum printing speed and avoiding overexposure effects.

**Table 1:** Parameters used to print the various regions of a reference cantilever (V1)

Region	Location	Slice size	Laser power	Writing speed
1	From 0 mm to 1.5 mm	100 µm	90%	30 mm/s
2	From 1.5 mm to 2.5 mm	50 µm	90%	30 mm/s
3	From 2.5 mm to 3.3 mm	20 µm	35%	6 mm/s
4	From 3.3 mm to 3.5 mm	10 µm	35%	5 mm/s

Regions are numerated incrementally from the base to the tip (Fig. 1c)

The total fabrication time for this cantilever design ranged from 3 h 30 min using the Dilase 3D HR model to 20 min with the latest model, the Dilase 3D MR (equipped with a double beam size, 5- and 20-µm x/y resolution, selected by using an automatic switch, and 100-mm/s maximal writing speed). After laser writing, the samples were immersed into two successive alcohol baths and dried with nitrogen. Figure 1e shows SEM images of a fabricated V1 cantilever.

Cantilevers with other shapes and geometries are presented in Figs. S1 and S2 (Supplementary Information). The V2 cantilever was similar to V1 with half dimensions (hence a 30 × 30 µm tip section). The V3 cantilever had the exact dimensions of V1, but it displayed a relatively sharp 60° angle tip. The V1x3 sample included three V1 cantilevers on the same cuboid base for parallelized spotting. Samples V1PL and V2PL were similar to V1 and V2 but had a gradually thickened backside (from 60 µm at the tip to 400 µm at the base for the V1PL version) used as a reinforcing strategy rather than the 2 front beam mechanical supports. Cantilevers V4 and V5 were V1-like but with different extremity shapes. V4 displayed a partially enclosed fluidic reservoir, while there was a circular recess at the tip of V5. Finally, sample V1M included a soft magnetic base that was fabricated using the DS-3000 polymer loaded with 6–8-µm-diameter Fe magnetic particles (Good fellow FE00-PD-000141, Iron-Powder; slice of 100 µm with 90% laser power and 30 mm/s writing speed). The cantilever was subsequently printed by changing the resist back to the previously used DS-3000 resist.

### Loading and spotting experiments

For spotting purposes, the fabricated polymer cantilevers were mounted onto a computer-controlled stage. The cantilevers were attached to a piezo-actuator allowing z displacement with submicrometric precision through a mechanical piece mounted on the piezo-actuator and double-sided adhesive tape. Loading reservoirs and spotting substrates were placed onto a stepper motorized stage allowing movements in the x, y and z directions. The spotting angle is the angle between the cantilever and the deposition surface; it was adjusted manually to 67°, 80° or 90° (±2°) according to the experiment via the mechanical piece mounted on the piezo-actuator. The overall loading and spotting processes were monitored through a Thorlabs DCC1545M-GL camera with a Navitar 1–51188 TenX zoom lens optical tube coupled to a ring light interface 2–50017. The loading was achieved by immersing the cantilevers in reservoirs containing a drop of the liquid to be deposited. Spotting was carried out by contacting the tips of the cantilevers with a dedicated surface for a specific duration. To this aim, loaded cantilevers were placed above the surface where droplets were meant to be delivered and were moved downwards in 1 µm increments until they touched the surface. The contact point was determined visually using the light reflected on the bending cantilevers upon contact, where drop formation could be clearly observed at high magnifications.

Various liquids were deposited onto microscope and gold-coated glass slides, all previously cleaned with acetone and deionized (DI) water. The wettability levels of the cleaned and gold-coated glass surfaces were determined by contact angle measurements using a GBX Digidrop contact angle meter to be 25 ± 2° and 81 ± 2°, respectively. The wettability of the polymerized DS-3000 constituting the cantilever was estimated to be approximately 60°. We used a mixture of 50% (v/v) DI water/glycerol and fluorescently labeled IgG molecules (goat anti-rabbit FITC conjugate and goat anti-mouse CF647 conjugate IgGs) diluted at 100 µg/mL in PBS with 5% (v/v) glycerol, a hydroxylated additive. Before the loading and spotting steps, the cantilevers were washed with acetone and DI water and dried by absorbing excess water with a paper towel. All spotting experiments were conducted at room temperature without controlling the humidity. The resulting droplet arrays were observed with an inverted Olympus microscope with 4X and 10X objectives, white light for water/glycerol droplets and appropriate filter cubes for fluorescent imaging of labeled antibody spots. Images were recorded with an ANDOR camera. Image analysis and curve fitting were conducted with ImageJ and Origin, respectively.

### Biofunctionalization experiments

Functionalization experiments were conducted by depositing droplets of thiolated oligonucleotides (ODN) onto aminosilanized glass slides (631–1550, VWR) coupled with a cross-linker. The slides were first cleaned using a Diener electronic Femto low-pressure plasma system (3 min, 75% O_2_, 25% Ar, 0.6 mbar pressure, and 80% generator power). The cleaned glass slides were immediately used for silanization in the liquid phase with 2.5% (v/v) (3-aminopropyl)triethoxysilane (APTES) (741442–100 ML, Sigma Aldrich) solution in anhydrous toluene (32249–1L-M, Sigma Aldrich). The glass crystallizer containing glass slides and aminosilane solution was stored in a desiccator with anhydrous CaSO_4_ desiccant for 5 h at room temperature (RT). To remove unbound APTES molecules, slides were subjected to a series of 2 min washing baths: anhydrous toluene, anhydrous ethanol (34852–1L-M, Sigma Aldrich), ethanol (96% pure, Carlo Erba reagents), and DI water (18 MΩ·cm). To finalize the reaction, slides were baked for 1 h at 110 °C. The silanized glass slides were incubated for 1 h with 0.1 mM N-γ-maleimidobutyryl-oxysulfosuccinimide ester (Sulfo-GMBS) cross-linker (22324, Thermo Fisher Scientific) in 7.42 pH 1x PBS (p4417–100TAB, Sigma Aldrich). The uncoupled Sulfo-GMBS was removed by rinsing slides with rinsing buffer (RB) (PBS 0.01 M, NaCl 0.537 M, KCl 2.7 mM, and 0.15% (v/v) Tween), filtered DI water, and dried with argon. The spotting step was performed at ambient temperature (25.4 ± 0.6 °C) and 16 ± 2% relative humidity (RH). The spotting cantilever was mounted with a CMOS camera (DigiMicro 2.0 Scale, Toolcraft, Conrad) on an x-y-z linear motorized stage (UTS100CC stage, Newport) that was computer-controlled using a 3-axis motion controller (ESP301, Newport). The cantilevers were rinsed with DI water and ethanol prior to use, and a 67 ± 2° spotting angle and a 250 ms contact time were used for drop delivery. The thiol-modified oligonucleotides (Zip1 and Zip2 sequences, Eurogentec, see Table S1 for the sequence description) were diluted at 10 µM in PBS with 50% (v/v) glycerol (G7757–1L, Sigma Aldrich) and spotted with the cantilever on two separated slides. The cantilever was properly washed with DI water and ethanol before and after spotting each thiol-modified ODN sequence. After spotting, the glass slides were stored inside a chamber filled with 50% (v/v) glycerol-water solution to maintain a constant humidity level. The chamber was stored at 25 °C for 3 h. Next, the slides were washed with RB, filtered DI water, and dried with argon. The slides were hydrated for 5 min with DI water and preblocked with RB for 5 min at room temperature (RT). Then, the surfaces were blocked with 1 wt.% bovine serum albumin (BSA) (98% purity, Sigma Aldrich) diluted in PBS for 30 min at RT, rinsed once with RB, and incubated with 0.2 µM of the complementary biotinylated strand of oligonucleotides (Zip1c and Zip2c, see Table S1) in hybridization buffer (PBS 0.005 M, NaCl 0.2685 M, KCl 1.35 mM, and 0.075% (v/v) Tween) for 15 min at 37 °C. The unhybridized complementary strands of ODN were removed by rinsing the slides several times with RB. Finally, the slides were incubated with 5% (v/v) streptavidin-R-phycoerythrin (SAPE) (S866, Thermo Fisher Scientific) in RB for 5 min in complete darkness for fluorescence detection. The slides were rinsed thoroughly with RB to remove unbound fluorescence dye. Fluorescence images were captured using an Olympus BX60 upright epifluorescence microscope (Hamamatsu CDD camera C5985–10, U-MWIG Olympus filter, and HBO 100 w/2 OSRAM mercury lamp).

## Results and discussion

### Printing fidelity

The replication fidelity of the printing technique is assessed by measuring characteristic features at the tip of a V1 printed cantilever (Fig. S3). Printed features are smaller than designed features by up to 15%. For instance, the 60 × 60 µm designed tip cross-section results in a printed 55 × 55 µm tip area, leading to a slightly smaller effective channel width (18.6 µm rather than 20 µm). However, these deviations are consistent with the x,y printing resolution of the machine (estimated to be 5 µm^27^).

### Loading and spotting studies

**Fig. 2:**
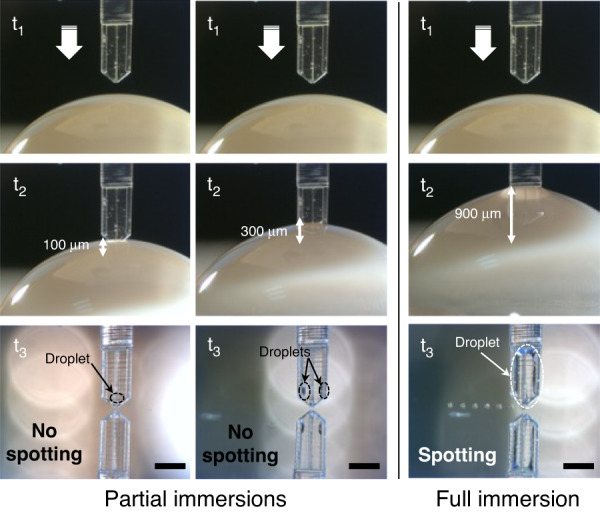
Cantilever loading process by immersion in a water/glycerol droplet. From top to bottom: sequential views of the loading process as the V1 cantilever moves down into the droplet and picture of the cantilever while spotting on a glass slide (scale bar = 500 µm). Left and center: cases where the cantilever is partially immersed (100 µm and 300 µm deep into the droplet from the cantilever tip). Right: full immersion (900 µm of the cantilever into the droplet)

We study the influences of the shape and geometry of the cantilevers on the loading and spotting capabilities. We first partially and fully immerse the V1 cantilever in loading drops of 50% (v/v) DI water/glycerol (Fig. S4b), where glycerol is used to reduce the solution evaporation rate, before spotting an array of droplets on a glass slide (Fig. 2). The cantilever is positioned vertically in these experiments, i.e., with a spotting angle of 90° relative to the substrate. We observe that as soon as the cantilever tip contacts the loading drop, the liquid fills the microfluidic channel. The latter behaves as an open microfluidic channel where the condition for spontaneous capillary flow is given by the following equation^36^:

1 $${{\cos }}\theta\, > \,\frac{{p}_{a}}{{p}_{l}}$$

where *θ* is the liquid contact angle, *p*_*a*_ is the length of the air−liquid interface perimeter and *p*_*l*_ is the length of the liquid−solid interface along the perimeter of the channel cross section, i.e., p_*a*_ = *w* and *p*_*l*_ = *w* + 2 *h* (where *h* is the channel depth and *w* is its width; Fig. S4a). For a rectangular section microchannel, such as the one used in this study, the ratio of the interface lengths is 1/6. Considering that the DS-3000 resist contact angle is <60°, the condition for spontaneous filling is expected. The dynamics of the capillary flow are recorded while loading a V1PL cantilever (Video S1). As shown in Fig. S5, the travel distance of the fluid is proportional to the square root of time (curve fitting results in an adjusted R-squared value of 0.996), indicating a viscous regime, where the fluid flow is governed by the balance between capillary forces and friction and can be described by the Lucas–Washburn–Rideal law^36^.

**Fig. 3:**
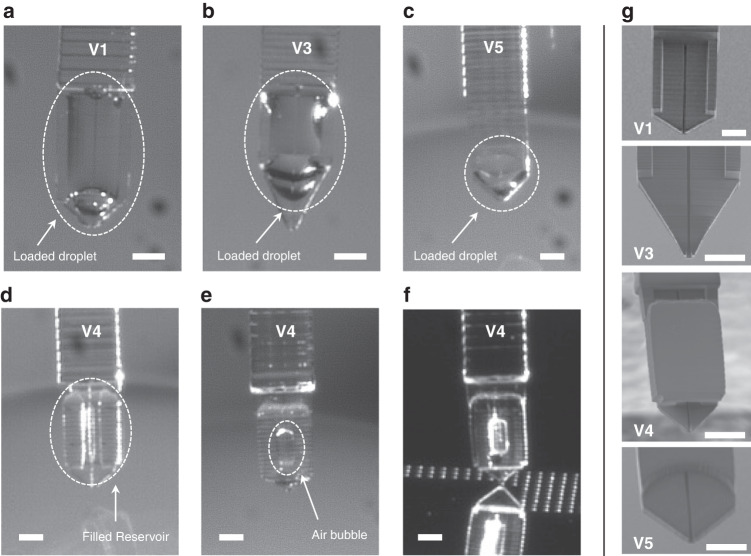
Loading various cantilevers by full immersion. **a**–**e** Optical photographs of the V1, V3, V5, and V4 cantilevers after loading by full immersion into a water/glycerol droplet showing the liquid remaining at the surface (scale bar = 200 µm). **f** Picture of the V4 cantilever during successful spotting (on a gold surface) despite the presence of an air bubble in the reservoir. **g** Images of the corresponding cantilever tips (scale bar = 200 µm)

Despite successful capillary filling of the channel of the V1 cantilever, we do not observe any drop formation upon contact of the cantilever with the surface. Partial immersion of the cantilever in the external loading reservoir leads to the presence of a drop on the surface of the cantilever and to erratic deposition. Whenever the drop loaded on the cantilever is pinned on the tip of the cantilever, it is transferred onto the surface upon direct contact, resulting in the deposition of a single large droplet; however, most of the time, when the loaded drop is located away from the tip, no droplet is delivered. However, after full immersion by plunging the entire portion of the cantilever that bears the strengthening beams, reproducible spotting of numerous liquid droplets is observed (Fig. 2). To tentatively explain this behavior, we consider the cantilever to be composed of an open reservoir (i.e., the droplet loaded on the cantilever surface) connected to an open microfluidic channel that reaches the cantilever tip (Fig. S4c). For drop delivery to occur by liquid transfer upon direct contact of the cantilever tip with the surface, the extremity of the cantilever must be wetted. In other words, the meniscus of the loaded droplet should be located in the vicinity of the tip extremity to favor contact wetting with the surface. We suspect that the concave meniscus pinned at the extremity of the channel does not offer proper wetting conditions for drop delivery. In the case of partial filling, which is when the channel is filled and a droplet is loaded at the surface of the cantilever upon partial immersion, the contributions of capillary pressure and Laplace pressure in the reservoir are insufficient to produce wetting conditions by acting on the meniscus at the tip of the cantilever if the loaded drop is located away from the tip. When this drop is pinned to the edge of the cantilever tip, wetting conditions enable liquid transfer; in that case, as observed in the dispensing of liquid through an apertured AFM probe, a liquid meniscus forms between the tip and surface where equilibrium is reached when the liquid meniscus fulfils the relationship between the equilibrium contact angles on the tip and substrate with a hydrostatic pressure imposed by the Laplace pressure of the reservoir^37^. Hence, since full immersion always results in the load of a bulging droplet pinned at the edges and apex of the cantilever, as seen on the bottom right photograph in Fig. 2, the wetting conditions are satisfied for the drop to always be delivered. Experiments conducted with other types of cantilevers have confirmed our assumptions. Successful channel loading of the V1PL cantilever is achieved, but no deposition occurs because its flat design does not promote droplet lading. In contrast, the presence of the beams favors the loading of large droplets onto the V3 cantilever upon full immersion. However, deposition fails again. As shown in Fig. 3b, the large beam-to-tip distance and the sharp design of the cantilever extremity result in positioning of the droplet meniscus far from the extremity of the cantilever. In summary, we observe that initiating and maintaining reproducible deposition are possible only when at least three of the following conditions are ensured: i) designing the cantilever with supporting beams to favor droplet loading, ii) performing full immersion of the tip during the loading step, and iii) setting a tip profile with an angle >45° to promote the placement of the drop meniscus close to the tip edge.

From these considerations, we design two extra cantilevers with dedicated reservoirs (Figs. S1 and S2). The first design, V4, includes a partially enclosed reservoir to minimize evaporation. The second design, V5, exhibits a circular topographical recess at the tip. When loading the V4 cantilever for the first time by full immersion, the liquid fills the whole reservoir, and spotting is successful. However, after performing liquid deposition and gradually emptying the reservoir by droplet delivery, all the subsequent loading by full immersion results in an air bubble trapped in the reservoir (Fig. 3e). This air bubble cannot escape the reservoir and does not prevent spotting.

**Fig. 4:**
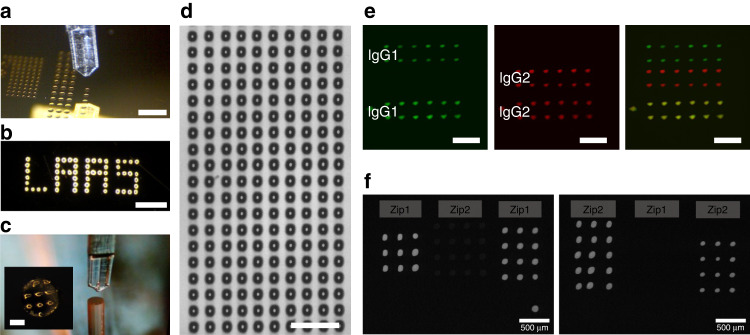
Spotting and biofunctionalization results. **a** Optical image of a polymer cantilever during drop deposition upon direct contact with a gold surface (scale bar = 500 µm). **b** Printed droplets on gold (scale bar = 500 µm). **c** Photograph of a V1 polymer cantilever while spotting on the extremity of a 300-µm-diameter gold-coated optical fiber bundle (the inset shows an array of deposited water/glycerol droplets: spotting time is 50 ms, center-to-center distance is 100 µm, scale bar is 100 µm). **d** Arrays of water/glycerol droplets deposited by a V1 cantilever with a contact time of 100 ms after a single load (scale bar = 300 µm). **e** Array of fluorescently labeled IgG molecules sequentially deposited by the 3D-printed cantilever (IgG1: goat anti-rabbit FITC-conjugated IgG, IgG2: goat anti-mouse CF647-conjugated IgG, and scale bar: 400 µm). **f** Fluorescence revelation with Zip1c (left) and Zip2c (right) of the spotted ODN microarrays

The loading of the V5 cantilever is achieved by dipping the cantilever sufficiently deep to ensure the immersion of the topographical feature, and droplets are successfully deposited on the glass slide. Since the circular feature is positioned very close to the cantilever tip (460 µm), this design offers the advantage of using a relatively small loading reservoir and of reducing the analyte consumed. In addition, the volume loaded on the V5 cantilever is noted to be approximately 5 nL (roughly estimated to be the volume of a drop of a perimeter equivalent to that formed by the tip and the circular feature on the cantilever surface with a contact angle of 60°), which is comparable to the volume loaded on the other cantilevers (e.g., approximately 5.6 nL in the reservoir of cantilever V4) and is much larger than the typical volume of a deposited droplet (250 pL for a 50 µm diameter droplet deposited on gold, with a contact angle of 80°). Hence, the use of an open reservoir at the tip of the cantilever, such as the one integrated into the V5 cantilever, provides enough liquid to print approximately 200 droplets. As a comparison, we deposit arrays of many droplets on an untreated gold-coated glass slide with a V1 cantilever, and we can deposit at least 225 droplets with a single load (Fig. 4d). Measurements conducted on 200 droplets indicate a mean drop diameter of 69.5 µm with a standard deviation of 2.7 µm, illustrating the high uniformity of drop size and deposited volume. Notably, this number of spotted droplets can largely be increased by the use of the V1x3 version of the MEMS spotting device that includes multiple cantilevers on a single base, exactly like the silicon version^9^.

We then study the influences of the contact time, spotting angle, surface wettability and cantilever dimensions on the sizes of the deposited water/glycerol droplets. The results are displayed on the graph of Fig. S6. The diameters of droplets deposited with the V1 cantilever with a spotting angle of 90° onto glass slides (contact angle of 25 ± 2°) increase greatly with contact time from ∼115 µm for a 50 ms contact time to ∼275 µm for a 5000 ms contact time. When positioning the cantilever at an angle of 80° with respect to the deposit surface, we observe that the diameters of the printed droplets are relatively large and that the increase with contact time is pronounced. We can explain this trend by the increase in apparent contact area when tilting the cantilever. We have printed droplets with the V2 cantilever that is half as small as the V1 cantilever, thus displaying a reduced tip section of 30 × 30 µm as opposed to 60 × 60 µm. Due to the smaller area contacting the deposit surface, droplets exhibit smaller diameters, ranging from ∼45 µm to ∼95 µm for 50 ms–5000 ms contact times when spotted on a glass surface at a 90° angle. In addition to the influences of the tip size and contact area between the cantilever and the deposit surface on the diameter of the spotted features, we observe the strong influence of the deposit surface wettability. Indeed, the diameters of droplets deposited with the V1 cantilever with a spotting angle of 90° on gold surfaces (contact angle: 81 ± 2°) only slightly increase from ∼55 µm to ∼65 µm with contact times ranging from 50 to 5000 ms. A similar trend, which is expected from the increased spreading of liquid onto hydrophilic surfaces, has previously been observed with Bioplume silicon cantilevers^10^, confirming the results presented herein. Notably, while elastic deformation of the cantilever is expected when pressing it down onto the surface during spotting, it does not experience major inelastic deformation, as illustrated by the high uniformity of the deposited droplets performed at high speed (2 droplets/cantilever/second, at least for a few hundred drops).

### Biofunctionalization studies

Next, since this tool is mainly devoted to the biofunctionalization of MEMS for biosensing applications, we fabricate simple protein and DNA microarrays using V1 cantilevers. First, two types of fluorescently labeled IgG molecules diluted in PBS (experimental section) are deposited onto a gold-coated glass slide. After depositing 2 rows of 6 droplets of goat anti-rabbit FITC-conjugated IgG, the cantilever is washed by sequentially dipping it in two reservoirs (where the cantilever is immersed 1 mm into the liquid from its tip), one containing water and one containing ethanol, for 5 s; we let the cantilever dry for 5 s after each immersion. Then, the cantilever is loaded with goat anti-mouse CF647 conjugate IgG, and 2 rows of 6 droplets are printed below the first array. The cantilevers are loaded with the goat anti-rabbit FITC conjugate IgG solution without conducting a washing procedure. An array of 2 rows of 6 droplets is then spotted below the goat anti-mouse CF647 conjugate IgG droplets. Figure 4e shows fluorescent images of the resulting microarray, where the images on the left and in the center are taken with filter sets designed to match the excitation/emission wavelengths of the FITC and the CF647 fluorophores. These two images are merged to produce the image on the right. As seen from these images, there is clear cross-contamination in the last printed array, where the presence of both fluorophores can be detected. However, the second array does not present any sign of cross-contamination, indicating the efficiency of the cantilever rinse. This washing protocol has been developed for the silicon version of the Bioplume spotter, and we confirm that it is suitable for the polymer version. Hence, one of the major advantages of using open fluidics on the cantilever lies in the simplicity and efficiency of the washing step, thus allowing the sequential deposition of multiple biomolecules with a single cantilever. We can use devices with multiple cantilevers, such as the V1x3 version, in combination with dedicated reservoirs to carry out multiplexed printing of various molecules, as previously demonstrated with the silicon Bioplume^14^.

We have fabricated simple DNA microarrays with thiolated oligonucleotides on aminosilanized glass substrates coupled with a heterobifunctional cross-linker (experimental section and Fig. S7). Two sequences of ODN are used as a proof-of-concept. On the first slide, two Zip1 arrays are spotted by a V1 cantilever, and a Zip2 array is deposited in the middle as a negative control. On a second slide, two arrays of Zip2 separated by a Zip1 array are printed by the cantilever. Fluorescence revelation is carried out with SAPE as described in the experimental section and in Fig. S8 after previously blocking the surface of the slide with BSA and incubating the samples with 0.2 μM complementary biotinylated ODN strands (Zip1c, which is complementary to Zip1, on the first slide and Zip2c, which is complementary to Zip2, on the second slide). The resulting fluorescence images are shown in Fig. 4f. The Zip1 patterns on the first slide exhibit a relatively intense level of fluorescence and reproducibility in the spot size (~100 μm), while the negative control, Zip2, shows a negligible fluorescence signal. Zip2 spots on the second slide have similar fluorescence levels, but no signal is detected on the negative control. These experiments demonstrate that the spotted molecules retain their biological functionality and high specificity.

As mentioned in the introduction, the silicon version of the Bioplume tool is used for various applications ranging from the fabrication of microarrays to the realization of optical polymer lenses for the collimation of vertical cavity surface emitting lasers. Nevertheless, this tool was originally developed for the biofunctionalization of fragile MEMS sensors, and it has been successfully used to deposit drops of molecularly imprinted polymers (MIP) on micromachined resonant membranes used for pesticide detection^13^. The Bioplume spotter has subsequently been used to functionalize objects with complex geometries, such as the extremity of nanostructured gold-coated optical fiber bundles to be used for remote biosensing via surface plasmon resonance (SPR)^38^. To optimize the SPR signals, the nanostructured fibers must display tapered fiber end profiles with truncated cone geometry (core diameter of 3–4 µm, micropillar base of 2–3 µm, and micropillar height of 7–10 µm) covered by a 50-nm-thin gold layer, raising issues when using silicon Bioplume technology. Indeed, the tips of the silicon cantilevers damage the sharp fiber extremities and the gold layer upon direct contact (Fig. S9). The use of flexible cantilevers fabricated by 3D printing using a resin with a Young’s modulus two orders of magnitude lower than that of silicon successfully solves this issue (Fig. 4c and Fig. S9)^39^.

#### Multimaterial printing for added functionality

**Fig. 5:**
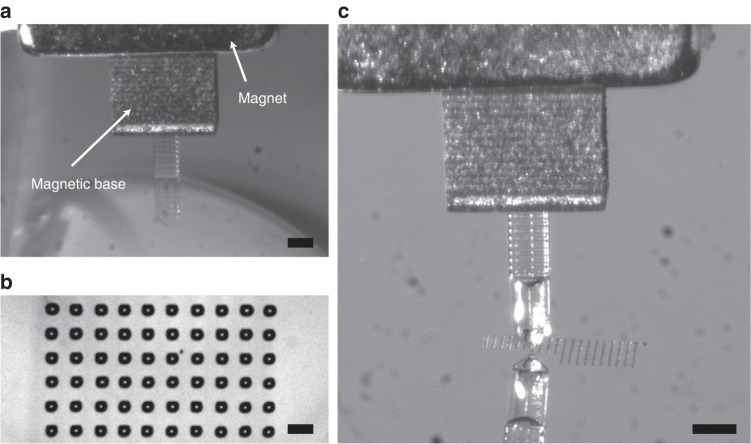
Magnetic droplet dispenser. **a** Optical photograph of the V1M cantilever held in the sample holder via a permanent magnet during loading. **b** Picture of an array of water/glycerol droplets spotted on a gold-coated slide produced by the V1M cantilever (scale bar = 100 µm). **c** Printing (scale bar = 500 µm)

Finally, we propose a strategy based on the successive 3D printing of various materials to fabricate a polymer cantilever with a magnetic base to simplify the sample manipulation and its positioning in the holder. The fabrication of the cantilever V1M is first conducted by filling the Dilase 3D tank with a DS-3000 resin loaded with 40%/w iron particles to create the base. Then, the sample is immersed in isopropanol for 5 min to remove the unpolymerized parts and dried with nitrogen prior to placing it back on the building platform of the printer. Finally, the hybrid resist is removed and switched to a DS-3000 tank to complete the fabrication of the cantilever directly onto its base (Fig. S2). The fabricated cantilever is then clamped to the sample holder by a 5-mm-side-length cubic NdFeB magnet (Fig. 5a); hence, rather than using double-sided adhesive tape to hold the cantilever chip, it is used to stick the magnet to the sample holder, obviating the risk of breaking the cantilever with tweezers upon detachment from the sticky tape. The V1M cantilever is loaded upon full immersion into a loading droplet, and droplets are printed onto a gold surface (Fig. 5c and Video S2). During printing, the cantilever does not seem to move, as indicated by the regularity of the spotted droplet array displayed in Fig. 5b, thus demonstrating the suitability of this clamping strategy. This composite cantilever is, to our knowledge, one of the few examples of a 3D-printed MEMS composed of various materials, making it possible to integrate complex functionalities.

## Conclusion

We have presented a MEMS droplet dispenser fabricated by stereolithography. This dispenser is based on the use of a microcantilever with an integrated microfluidic channel and reservoir that delivers tens of microscale diameter droplets upon direct contact of the cantilever tip with a surface. The use of 3D printing for this specific application offers numerous advantages, such as facile, affordable and fast manufacturing, that allows rapid prototyping with design flexibility; with this process, various shapes and dimensions are easily accessible, and it is possible to use various polymers. We have taken advantage of the two latter characteristics to design various cantilevers used to study the influence of the cantilever shape on the success of drop delivery, and we have integrated a magnetic base to facilitate the handling of the device. The biofunctionalization capability of the presented tool is demonstrated by fabricating antibody and DNA microarrays. The spotting device can print up to 200 highly uniform drops in a single load with a maximum speed of 2 droplets/cantilever/second; notably, multiple cantilevers can be integrated on the same chip to increase the throughput. The diameter of printed droplets ranges from ∼50 µm to >300 µm to date, and upon fabrication of smaller cantilevers using advanced lithography techniques, e.g., two-photon polymerization, this drop size can be decreased. We believe that this work shows that stereolithography is a powerful tool for the alternatively fabrication of low-cost MEMS devices with high design flexibility and that it can increase the accessibility of MEMS technology.

## Supplementary information

Supplementary Information

Video S1

Video S2
