# Supplementary material for: Rapid prototyping of a polymer MEMS droplet dispenser by laser-assisted 3D printing
Source: Microsyst Nanoeng. 2023 Jul 4;9:85. doi: 10.1038/s41378-023-00559-3 (PMC10318032; doi:10.1038/s41378-023-00559-3)
Supplement: Supplementary file 1 — Supplementary Information [file 41378_2023_559_MOESM1_ESM.pdf]

Supplementary Information

Rapid prototyping of a polymer MEMS droplet dispenser by laser-assisted 3D printing

Rémi Courson[1], Oleksii Bratash[2], Ali Maziz[1], Cloé Desmet[2], Ricardo Alvarado Meza[2], Loïc Leroy[2], Elodie Engel[2], Arnaud Buhot[2], Laurent Malaquin[1], and Thierry Leïchlé[1,3]

[1] LAAS-CNRS, Université de Toulouse, CNRS, 31400 Toulouse, France
[2] Université Grenoble Alpes, CNRS, CEA, IRIG, SyMMES, 38000 Grenoble, France
[3] Georgia Tech−CNRS International Research Laboratory, Atlanta, Georgia 30332, United States

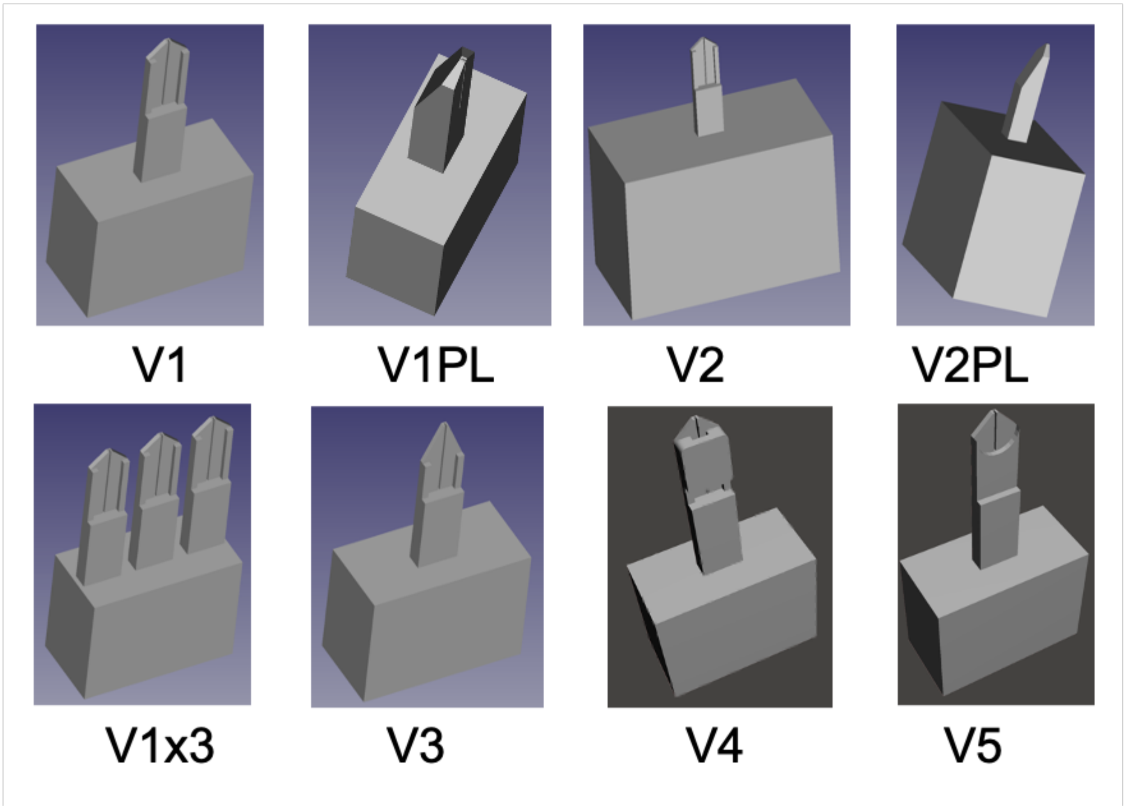

Figure S1. Cantilevers of various geometries fabricated and tested. V1 is the reference cantilever of following dimensions: 2 mm in length, 500 µm in width, 200 µm and 60 µm in thickness at the base and at the extremity; the sharpened extremity forms a 45° angle with a 60 µm x 60 µm tip cross-section. It includes a one-sided open, 1 mm long, 20 µm wide microfluidic channel with a section of 50 µm x 20 µm at the cantilever tip. It includes two strengthening beams. V2 is similar to V1 with half dimensions. V3 has a sharper 60° angle tip. V1x3 includes 3 V1 cantilevers. V1PL and V2PL have a gradually thickened backside. V4 displays a partially enclosed fluidic reservoir (of 160 x 580 x 60 µm^3 for an inner volumes of 5.6 nL). V5 exhibits a circular topographical recess at the tip (with a diameter of 460 µm).

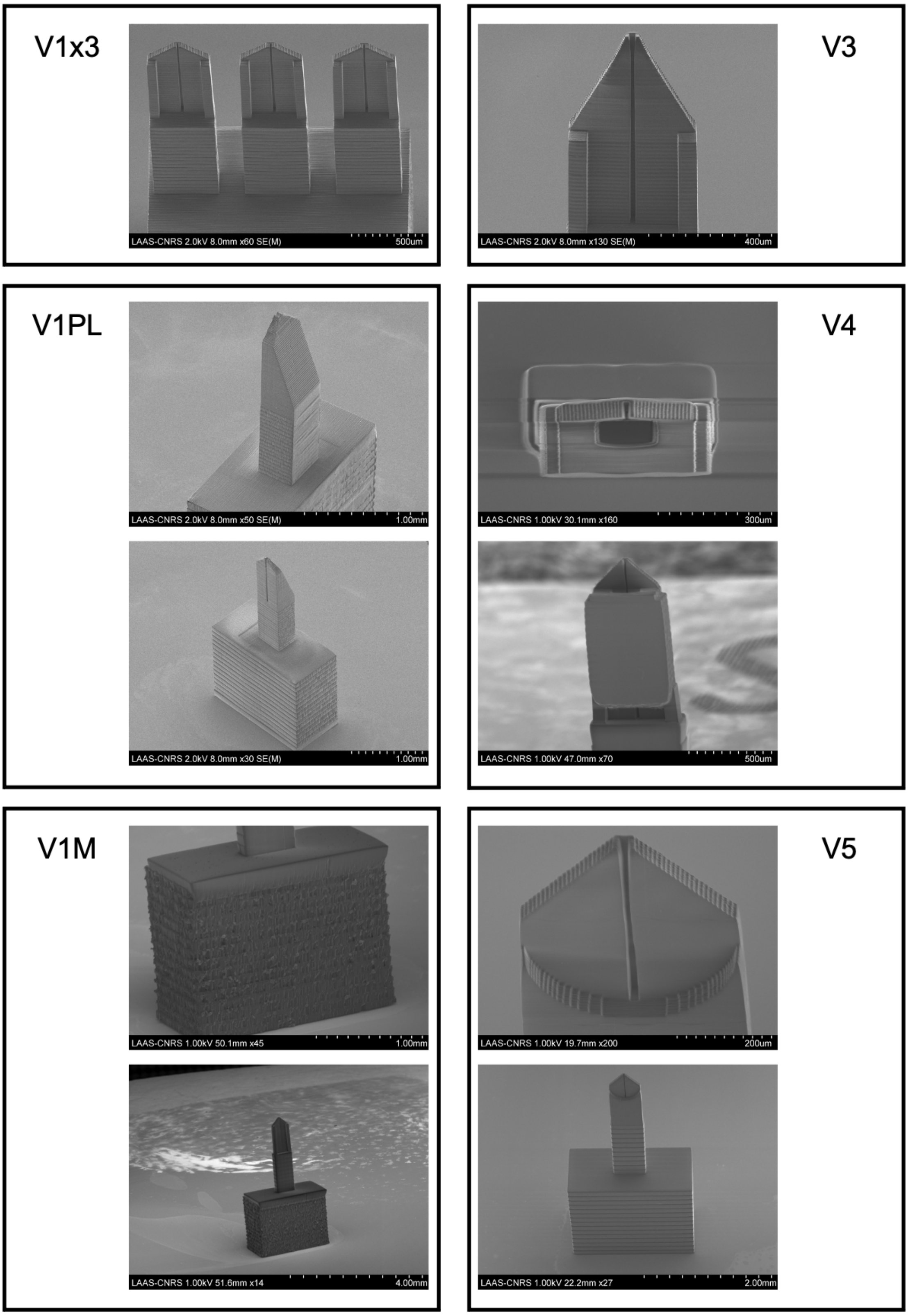

Figure S2. SEM images of the 3D printed polymer cantilevers of various shapes and geometries.

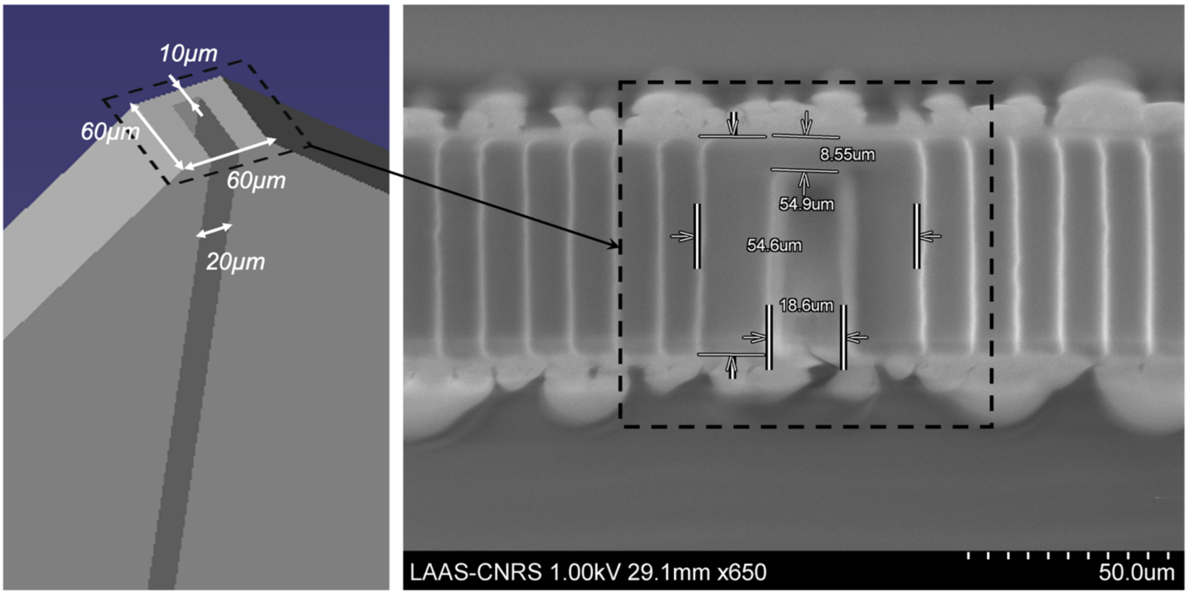

Figure S3. Comparison of pattern sizes of the designed (schematics, left) and printed cantilever tip (SEM image, right).

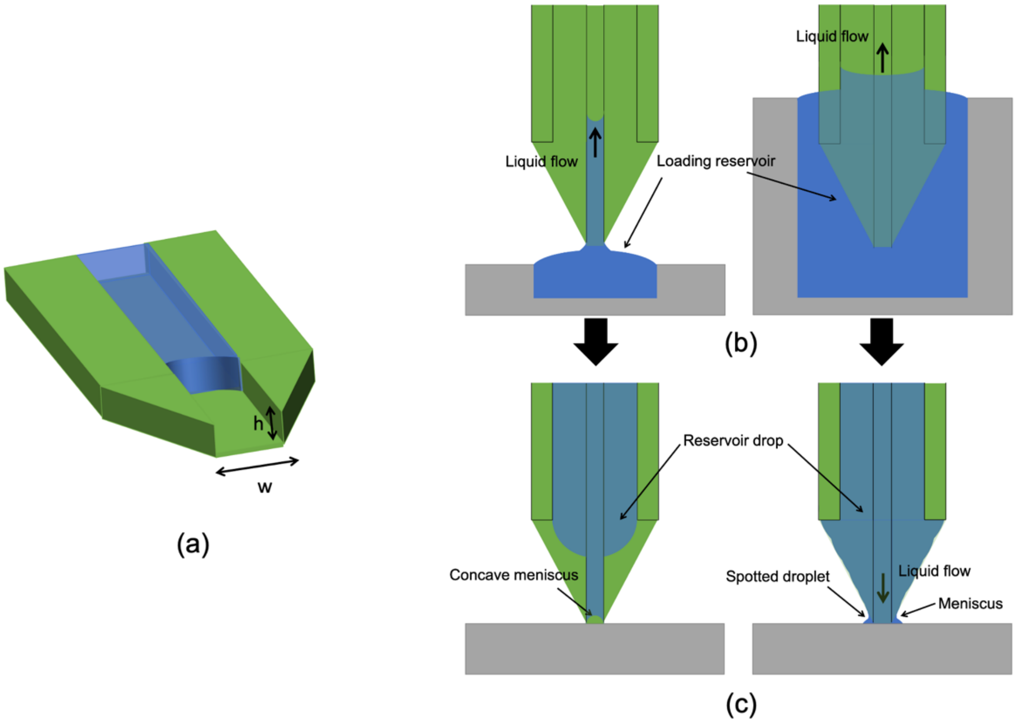

Figure S4. (a) Schematics of the cantilever tip showing the dimensions of the integrated fluidic channel. (b) Schematics depicting the liquid flow during the loading step: either by dipping the cantilever tip (left) or the reservoir of the cantilever (right). (c) Schematics of two observed spotting configurations: when the reservoir drop is located away from the tip of the cantilever (left), when the loaded drop used as a reservoir is pined on the edge of the cantilever (right). Drop deposition seems to occur only in the latter case.

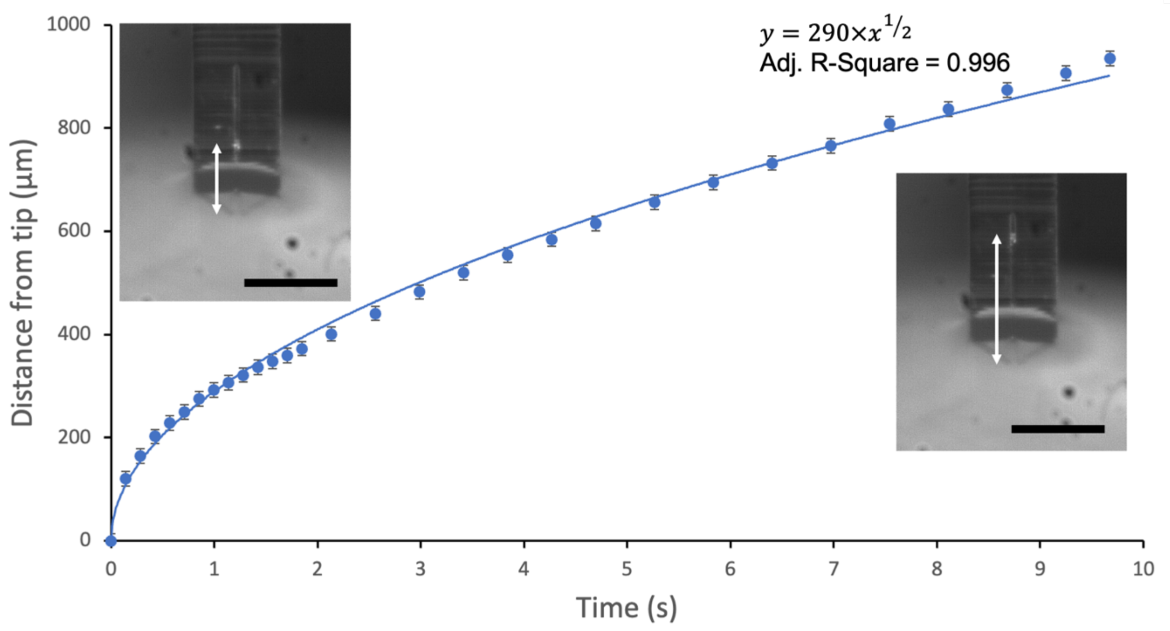

Figure S5. Travel distance of the liquid from the tip of the cantilever in the microfluidic channel as a function of time during the loading of the V1PL cantilever with 50% (v/v) water/glycerol mixture (dots: experimental data; line: polynomial fit). The insets show photographs of the cantilever during the loading process at two different times where the white arrows show the distance travelled by the liquid (scale bar = 500 µm).

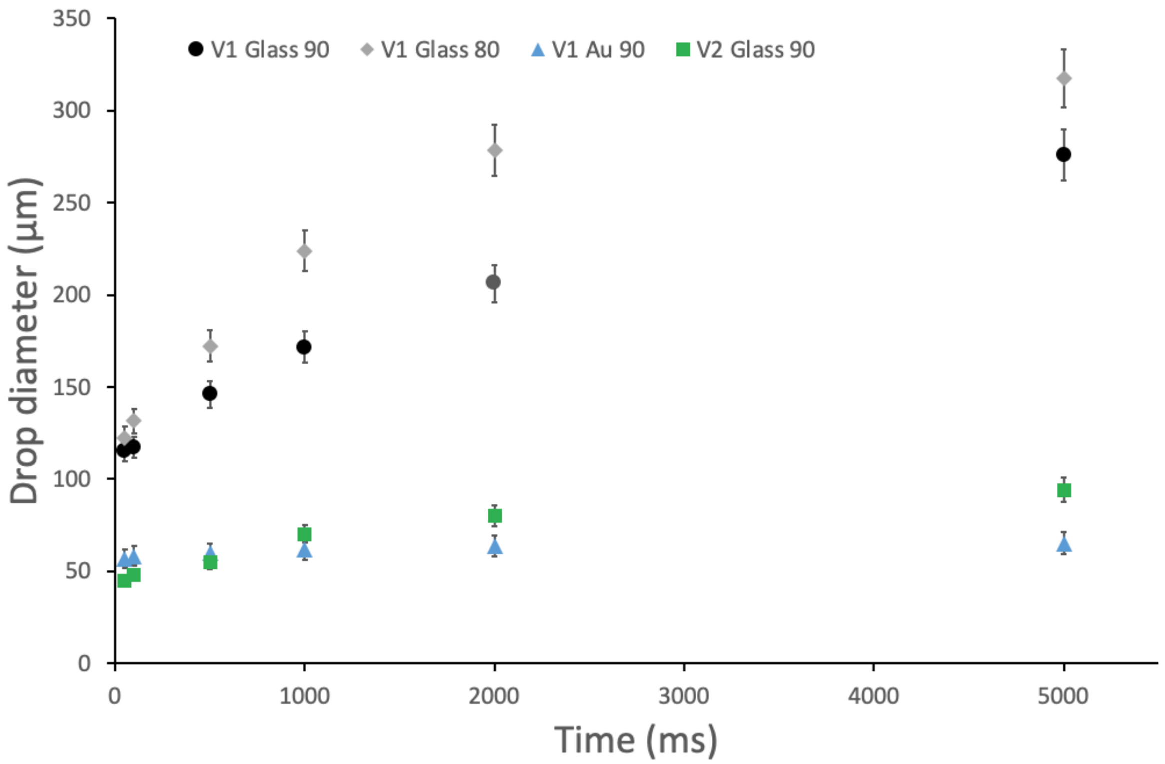

Figure S6. Diameter of the deposited droplets (50% (v/v) water/glycerol) as a function of spotting time for various designs of cantilevers (V1 and V2) on two types of surfaces (glass and gold) with different spotting angles (80° or 90°). Each point corresponds to a single droplet and the error bars account for the measurement error when manually estimating the drop diameter with Image J.

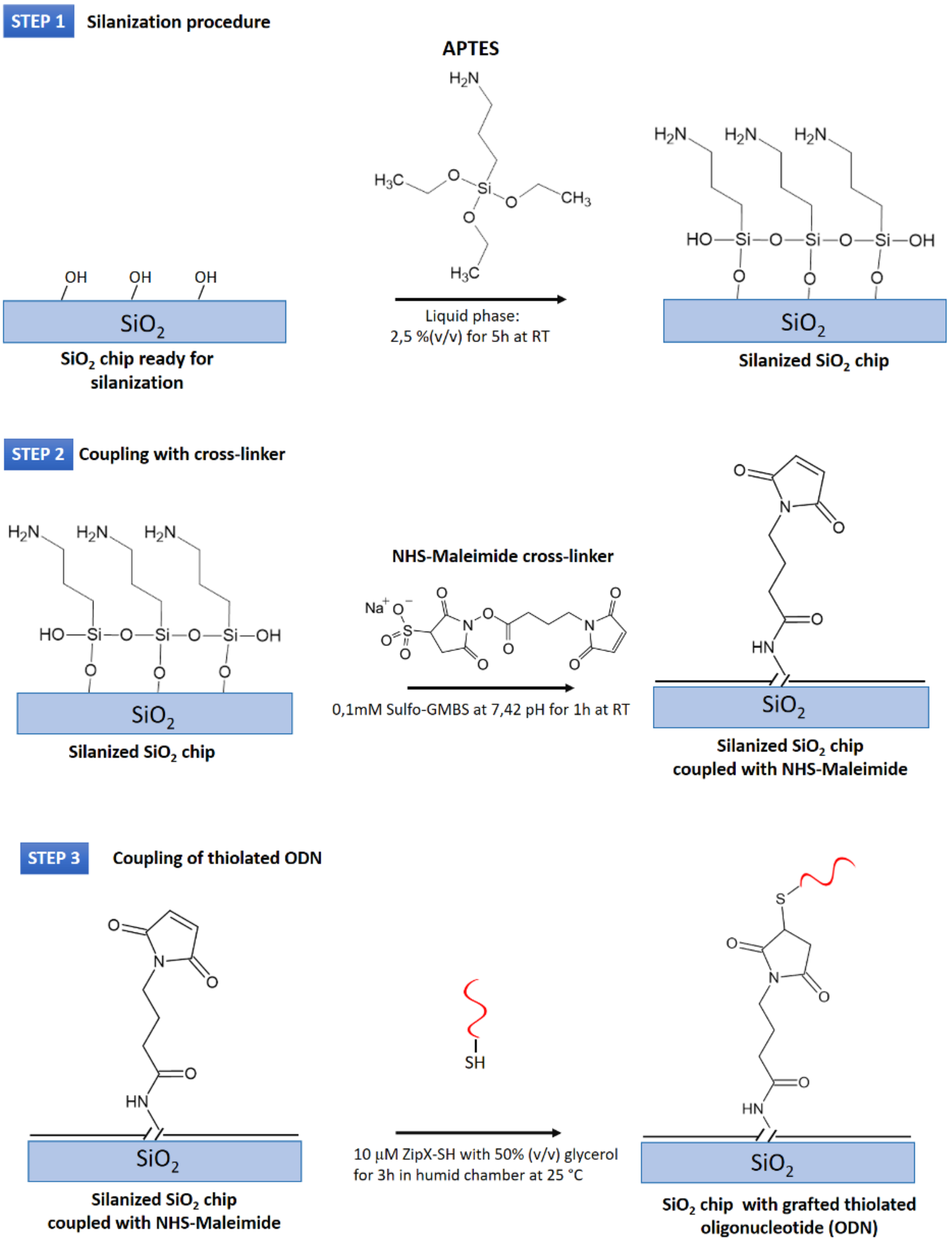

Figure S7. Schematic representation of the ODN microarray fabrication.

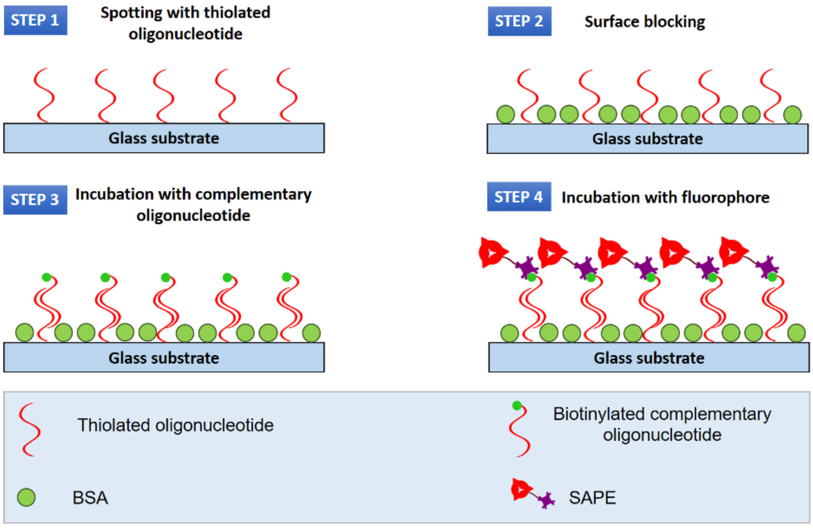

Figure S8. Schematic representation of steps during fluorescence revelation.

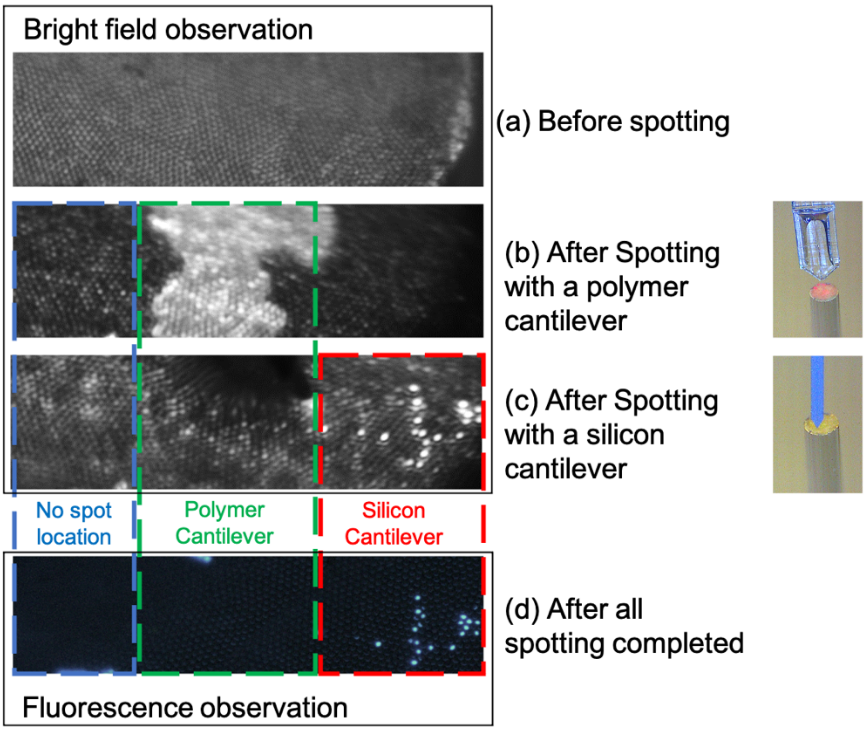

Figure S9. Optical images showing spotting results onto the extremity of a nanostructured gold-coated (50 nm) optical fiber bundle. The experiments were conducted with silicon and polymer cantilevers and a 50% (v/v) glycerol solution. Comparison of surface state before spotting (a) and after each spotting step (b), (c) imaged with a USB-camera. Blue selection: unspotted area; Green selection: polymer cantilever spotted area (showing a deposited droplet); Red selection: silicon cantilever spotted area (no droplet deposition). Note: the droplet deposited with the polymer cantilever has dried out before conducting deposition with the silicon cantilever. (d) Fluorescence image of the gold-coated face obtained with an inverted microscope where fluorescence originates from the germanium oxide optical fiber excited by UV light. Light (blue) coming through the gold-less (*i.e.* damaged) areas demonstrates that, unlike the polymer cantilever, the silicon cantilever damages the gold layer.

Table S1. Oligonucleotide sequences used for the experiment. “Thiol” indicates the thiol modification, while “Bio” represents biotin attached to ODN sequence.

**Name**	**Sequence 5’ → 3’**
Zip1	Thiol-TTT-TTT-TTT-TGA-CCG-GTA-TGC-GAC-CTG-GTA-TGC-G
Zip2	Thiol-TTT-TTT-TTT-TGA-CCA-TCG-TGC-GGG-TAG-GTA-GAC-C
Zip1c	Bio-CG-CAT-ACC-AGG-TCG-CAT-ACC-GGT-C
Zip2c	Bio-GG-TCT-ACC-TAC-CCG-CAC-GAT-GGT-C

Video S1. Recorded real-time video showing the dynamics of the capillary flow in the microchannel while loading a V1PL cantilever by dipping the tip of the cantilever.

Video S2. Recorded real-time video showing the spotting of an array of droplets by a V1M cantilever onto a gold surface.
